# Cytochrome P450 in living donor liver transplantation

**DOI:** 10.1186/s12929-015-0140-4

**Published:** 2015-05-15

**Authors:** King-Wah Chiu, Toshiaki Nakano, Kuang-Den Chen, Li-Wen Hsu, Chia-Yun Lai, Ching-Yin Huang, Yu-Fan Cheng, Shigeru Goto, Chao-Long Chen

**Affiliations:** Liver transplantation program, Kaohsiung Chang Gung Memorial Hospital, Chang Gung University, College of Medicine, 123 Ta-Pei Road, Niao-Sung District, Kaohsiung 833 Taiwan

**Keywords:** Cytochrome P450, CYP2C19, CYP3A4, CYP3A5, MDR1, Living donor liver transplantation, Pyrosequencing, Homogenous phenomenon

## Abstract

Cytochrome P450 metabolizes many drugs in the liver. Three genotypes of CYP2C19 with extensive, intermediate, and poor metabolizing activity, respectively, have been identified in peripheral blood of transplant recipients and new liver grafts in living donor liver transplantation (LDLT). The expression of the final genotype in liver graft biopsies depends on the donor, whereas the expression in peripheral blood mononuclear cells depends on the recipient. The metabolizing isoenzyme of the major anti-rejection agents passes through CYP3A4, CYP3A5 and MDR1, which have also been identified to have similar biological characteristics as genotype of CYP2C19 in liver tissue. Recently, pyrosequencing has been used to investigate the expressions of different genotypes in liver grafts in LDLT. This review focuses on recent findings regarding the biological expressions of the CYP2C19, CYP3A4, CYP3A5 and MRD1 genotypes in liver grafts before and after LDLT. The application of pyrosequencing may be beneficial in further research on liver transplantation. Laser capture microdissection of hepatocytes in liver grafts may be a direction for future research.

## Core tip

The genotypic expressions of CYP2C19, CYP3A4, CYP3A5 and MDR1 contribute to the mainstream by donor liver grafts in living donor liver transplantation. Pyrosequencing can identify homogenous phenomenon when different genotype expressions are found between the donor and recipient. These findings can help transplant hepatologists and those in the field of organ transplantation to understand how drug metabolism modifies cytochrome P450 in a new graft after liver transplantation.

## Introduction

Cytochrome P450 contain isoenzymes which metabolize many drugs in the liver [1]. To understand this complicated system, CYP2C19 and its genotypes can be used to describe the characteristics of the isoenzymes in cytochrome P450. CYP2C19 is one of the important isoenzymes responsible for drug metabolism including anti-convulsant and antacid agents [2, 3]. Based on the metabolic rate of a drug, there are three genotypes including extensive metabolizer (homozygous extensive metabolizer, HomEM), intermediate metabolizer (heterozygous extensive metabolizer, HetEM) and poor metabolizer (PM). The distribution of each differs depending on ethnicity. We previously identified that PM accounts for about 13.3 to 16.7 % of cases in Taiwan [4, 5]. In liver transplant recipients with the PM CYP2C19 genotype, lower doses of proton-pump inhibitors can be used to avoid drug intoxication in the treatment of peptic ulcer disease [6]. However, changes in the genotype in recipients and donors such as HomEM to PM or PM to HetEM in the setting of living donor liver transplantation (LDLT) have yet to be elucidated. This review focuses on our recent reports to investigate the isoenzymes that are modified by cytochrome P450 after LDLT.

## Reviews

### CYP2C19

CYP2C19 is one of the polymorphic isoenzymes in the cytochrome P450 system that is responsible for the metabolism of several therapeutically important drugs such as proton-pump inhibitors including omeprazole, lansoprazole and pantoprazole [7]. Because of the impact on drug metabolism, CYP2C19 may be important in LDLT. We previously used DNA extraction and polymerase chain reaction (PCR) to amplify DNA with all six fragments of the CYP2C19 gene corresponding to exon 1 (406 bp), 2–3 (606 bp), 4 (271 bp), 5 (409 bp), 7 (325 bp), and 9 (529 bp). Genomic DNA was isolated from the peripheral blood mononuclear cells of donors and recipients, and the CYP2C19 polymorphisms were identified. HomEM had wild-type alleles (*1/*1), HetEM had one mutated allele (*1/*2 or *1/*3), and PM had homozygous mutations (m1 in exon 5 or m2 in exon 4) of CYP2C19 (*2/*2, *3/*3 or *2/*3). Although the genotypes of CYP2C19 were different between the donors and recipients before LDLT, the original genotype expressions in the peripheral blood of the recipients did not affect the CYP2C19 genotypes of the donor liver grafts with different allelic patterns after LDLT [4].

In fact, the liver enzymes were easily detected early after LDLT, and the one of the important concerns was acute rejection. The incidence of acute rejection was 12.9–15.0 % in our earlier reports [5, 8]. When elevated liver enzymes are noted within 1 month after liver transplantation and the ratio of aspartate transferase/alanine transferase (AST/ALT) is less than 1.0, it should be considered an episode of acute rejection [8]. Many interesting findings have been reported when the genotypes of CYP2C19 have been applied to abnormal liver enzymes after LDLT. For example, the incidence of abnormal liver enzymes has been reported to be higher with CYP2C19 genotype HomEM (84.2 %) than HetEM (51.5 %) and PM (16.7 %), with a linear trend relationship [5]. Logistic regression analysis showed that the higher risk estimate of abnormal liver enzymes with HomEM was 26.7 times that of PM and HomEM 5 times that of PM [5]. This suggests that recipients with the HomEM CYP2C19 genotype may have a higher ability to metabolize drugs and therefore require a higher dose of immunosuppression agents after LDLT.

**Fig. 1:**
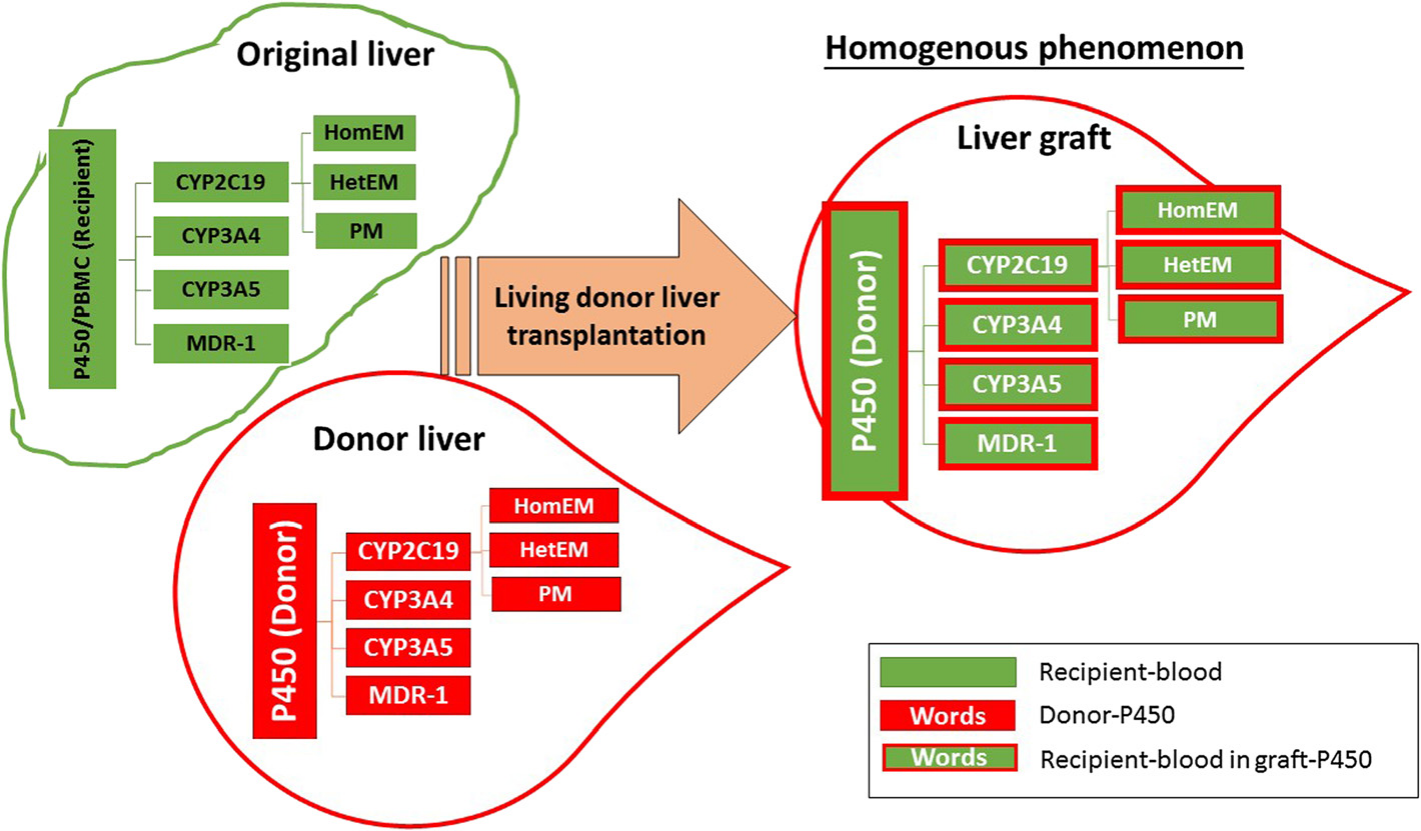
Picture of the homogenous phenomenon arising from the different genotypes of CYP2C19, CYP3A4, CYP3A5 and MDR1 between the donor and recipient in the cytochrome P450 system after living donor liver transplantation. The homogenous phenomenon is representing with the recipient blood (green block) go in the liver graft (white color) but the black ground of cytochrome P450 is still remain their own characteristic of isoenzymes even the recipient blood go through the graft

Percutaneous liver biopsies are performed when clinically required after LDLT. The polymorphisms of CYP2C19 found in liver tissue depends on the liver graft of the donor, even if the genotype is different between the recipient and donor. Western blotting can be used to identify the genotype, however the reason for the discrepancies between extensive and poor metabolizers in CYP2C19 after LDLT have yet to be explained [9]. In our previous study, direct sequencing of CYP2C19 genotypes showed that the sequence of graft liver CYP2C19 at exon 5 (681G > A) expressed a mixed pattern but was finally similar to that of the donor after LDLT. We named this morphological change the homogenous phenomenon [10] as shown in Fig. 1. Although the peripheral blood continuously flows through the liver graft, the effect on genotype expression from the recipient was limited. In DNA sequencing, the final presentation of CYP2C19 polymorphisms of the liver graft fully expressed the genotyping characteristic arising from the donor with limited morphological changes. In a donor with the CYP2C19 genotype PM (m1 in exon 5, 681A, *2/*2) and a recipient with HetEM (681 A/G, *1/*2) LDLT, follow-up liver biopsies showed a mixed pattern of CYP2C19 sequencing with a similar wave pattern but smaller amplitude than that of the donor [10]. It is known that the major anti-rejection agents such as cyclosporine A and tacrolimus are metabolized through cytochrome P450 with isoenzymes CYP3A4, CYP3A5 and multiple drug resistant 1 (MDR1), however CYP2C19 may not be involved with those agents. Therefore, we performed further research on CYP3A4, CYP3A5 and MDR1.

### CYP3A5

CYP3A5 is one of the most important isoenzymes for the metabolism of anti-rejection agents in cytochrome P450. Different from CYP2C19, CYP3A5 does not have any other genotypes. The morphological pattern did not present a significant quantitative expression in direct DNA sequencing or Western blotting. Investigations of CYP3A5*3 haplotypes with A/A, A/G and G/G genotypes may lead to a better understanding of the possible homogenous phenomenon in LDLT. We used PCR assays with the forward primer 5′-CATGACTTAGTAGACAGATGAC-3′ and reverse primer 5′-GGTCCAAACAGGGAAGAAATA-3′, and PCR with restriction fragment length polymorphism (PCR-RFLP) to explore the positions of CYP3A5 polymorphisms at rs776746 and identified the haplotypes of a single nucleotide polymorphism (SNP). The CYP3A5 *1/*1 genotype showed bands of 148, 125 and 20 bp in length, and the CYP3A5 *3/*3 genotype showed bands of 168 and 125 bp in length [11].

Pyrosequencing was performed with PyroMark Assay Design Software 2.0, and PCR assays were prepared with a PyroMark PCR Kit (Qiagen). The biotinylated forward primer 5′-TGTACCACCCAGCTTAACGA-3′ and a reverse primer at the 5′ end (5′-GGTCCAAACAGGGAAGAAAT-3′) were applied. The biotinylated PCR products were analyzed and expressed with proportional haplotyping of CYP3A5*3 from the peripheral blood and liver graft biopsy tissue of the recipients who underwent LDLT [11]. This method allowed for the objective investigation of the proportions of A/A, A/G and G/G and comparisons before and after LDLT on changes in cytochrome P450. RFLP was also performed to document the products belonging to A/G or G/G when an unusual interpretation was noted in pyrosequencing. According to pyrosequencing of the CYP3A5*3 genotypes, the homogenous phenomenon could be identified in the liver graft of the recipient after LDLT. This suggests that the final expression of CYP3A5*3 in liver grafts is truly dependent on the donor with only a limited change in the recipient. This biogenetic effect could potentially be predicted before LDLT in the screening stage of liver donation.

### CYP3A4

Similar to the CYP3A family, CYP3A4*18 could be easily identified with PCR-RFLP for DNA sequencing as with CYP3A5*3. A different primer for the PCR assay was used with a forward primer 5′-CACCCTGATGTCCAGCAGAAA CT-3′ and reverse primer 5′-AATAGAAAGCAGATGAACCAGAGCC-3′. The probe was prepared with 5′-TTTTTTTTTTTTTTT TTTTTTTTTACCTCCTCCCTCCTTCTCCATGTAC-3′ for CYP3A4*18-G and 5′-TTTTTTTTTTTTTTTTTTTTTTACCTCCTCCCTCCTTCTCC ATGTAT-3′ for CYP3A4*18-A [12]. Based on the haplotypes of the SNP from PCR-RFLP, three polymorphism genotypes were expressed as T/T, T/C and C/C by CYP3A4*18. CYP3A4 is an important isoenzyme in cytochrome P450 in liver and renal transplantation. Particular attention to the role of CYP3A4 when using anti-fungal agents such as ketoconazole concomitantly with anti-rejection drugs such as tacrolimus should be paid as this can cause severe pharmacokinetic drug to drug interactions [13–15]. In the CYP3A4*18 genotyping pyrosequence of the liver graft biopsies, the homogenous phenomenon was identified with the CYP3A4*18 SNP with the wild-type T allele and mutant variant C allele of the liver grafts, which arose from the donor and was different from the peripheral blood of the recipient after LDLT. The clinical implication is that recipients may be need a high dose of tacrolimus due to the extensive metabolism of the potential donor expressing CYP2C19 HomEM and CYP3A4*18 exon 10 wild-type 878 T > C/T after liver transplantation.

### MDR1

Transporter proteins (MDR1, gene coding glycoprotein-P, P-gp, ABCB1 gene) impact the required dose of anti-rejection organ transplantation agents such as tacrolimus and cyclosporine A due to pharmacokinetics. MDR1-3435 is a common polymorphism involved in both the pharmacokinetics and pharmacodynamics in LDLT. We used PCR-RFLP to explore genotype exon 26 C3435T variant alleles in MDR1, and PCR assays using the forward primer 5′-TGCTGGTCCTGAAGTTGATCTGTGAAC-3′ and reverse primer 5′-ACATTAGGCAGTGACTCGATGAAGGCA-3′. The PCR product was digested with Sau3A I. In the pyrosequencing expression of the SNP, the C allele was the wild-type, and the T allele was the mutant variant of MDR1-3435. The homogenous phenomenon was also identified in the difference between the liver graft and the peripheral blood of the recipients in C > C/T, C/T > C, C/T > T and T > C/T, but was similar to those of the donors after LDLT [16]. The genetic polymorphisms of the drug-metabolizing enzymes was found to be different from the donor, and a homogenous phenomenon was identified. However, changes in isoenzyme polymorphisms in the liver grafts were not manifested in the peripheral blood. Therefore, to understand the correlation of genes in liver grafts after liver transplantation when liver graft biopsy tissue is available from the recipient, the drug concentration in the blood is important, however examinations of the donor blood can further reveal the association between the liver graft and recipient after LDLT. It is difficult to know whether anti-rejection agent metabolism occurs through the isoenzymes of the liver graft, or whether it is related to intestinal isoenzymes. Small expressions of intestinal isoenzymes have been reported [17–19]. In our recent report, we investigated the relationship between CYP2C19 and CYP3A4*18, CYP3A5*3, and MDR1-3435. None of the haplotypes of these isoenzymes had a significant difference on the proportional distribution [16]. In other studies, liver graft hepatocytes were isolated using laser capture microdissection [20–22]. Interestingly, two different genotypes of isoenzymes were identified in the cytochrome P450 system, one arising from the donor liver and the other originating from the recipient’s circulating blood. The drugs passing through the common polymorphism may impact both the pharmacokinetics and pharmacodynamics. Finally, acute rejection usually occurs within one month after liver transplantation, and the homogenous phenomenon may play a role in the slowdown of acute rejection after LDLT. Our findings also suggest that intestinal isoenzymes play a compensatory role within one month after liver transplantation [13, 23]. With time, the coordinator role of the different genotypes creates a homogenous phenomenon to adjust the anti-rejection agents. Therefore, acute rejection should be resolution about one month after LDLT [24, 25].

In other words, CYP2C19 is one of the common isoenzymes of cytochrome P450 in the field of the gastroenterology for the proton pump inhibitor metabolism. The characteristic of different metabolism is believable treatment guide line by the gastroenterologists in clinical practice. In our recent reports, the genotypes of CYP2C19 could be modified by the donor graft not only on the drug metabolism for the peptic ulcer, but also possible on the immunosuppression agent causing abnormal liver function at the first month after LDLT. It may be play a major role to the monitoring of the drug metabolism in immunosuppression effect. We step-by-step to study more and more isoenzymes related to the field of the liver transplantation and discovery the evidence of homogenous phenomenon similarly take place on the CYP3A4, CYP3A5 and MDR-1. This information should be benefit on the pharmacologic and organ transplantation worldwide.

## Conclusion

In the cytochrome P450 system, isoenzyme polymorphisms may play an important role in drug metabolism, primarily in donor liver grafts with different genotypes after liver transplantation. Genomic polymorphisms may be mutant with a biogenetic phenomenon, and the mainstream expression depends on the donor in the cytochrome P450 system. Pyrosequencing may be a reliable method to identify changes in polymorphisms after LDLT.
